# Establishment of feline intestinal epithelial cell cultures for the propagation and study of feline enteric coronaviruses

**DOI:** 10.1186/1297-9716-44-71

**Published:** 2013-08-21

**Authors:** Lowiese MB Desmarets, Sebastiaan Theuns, Dominique AJ Olyslaegers, Annelike Dedeurwaerder, Ben L Vermeulen, Inge DM Roukaerts, Hans J Nauwynck

**Affiliations:** 1 Department of Virology, Parasitology and Immunology, Faculty of Veterinary Medicine, Ghent University, Salisburylaan 133, B-9820, Merelbeke, Belgium

## Abstract

Feline infectious peritonitis (FIP) is the most feared infectious cause of death in cats, induced by feline infectious peritonitis virus (FIPV). This coronavirus is a virulent mutant of the harmless, ubiquitous feline enteric coronavirus (FECV). To date, feline coronavirus (FCoV) research has been hampered by the lack of susceptible cell lines for the propagation of serotype I FCoVs. In this study, long-term feline intestinal epithelial cell cultures were established from primary ileocytes and colonocytes by simian virus 40 (SV40) T-antigen- and human Telomerase Reverse Transcriptase (hTERT)-induced immortalization. Subsequently, these cultures were evaluated for their usability in FCoV research. Firstly, the replication capacity of the serotype II strains WSU 79–1683 and WSU 79–1146 was studied in the continuous cultures as was done for the primary cultures. In accordance with the results obtained in primary cultures, FCoV WSU 79–1683 still replicated significantly more efficient compared to FCoV WSU 79–1146 in both continuous cultures. In addition, the cultures were inoculated with faecal suspensions from healthy cats and with faecal or tissue suspensions from FIP cats. The cultures were susceptible to infection with different serotype I enteric strains and two of these strains were further propagated. No infection was seen in cultures inoculated with FIPV tissue homogenates. In conclusion, a new reliable model for FCoV investigation and growth of enteric field strains was established. In contrast to FIPV strains, FECVs showed a clear tropism for intestinal epithelial cells, giving an explanation for the observation that FECV is the main pathotype circulating among cats.

## Introduction

Feline coronaviruses (FCoVs) are associated with both enteric and systemic diseases in domestic and wild *Felidae*. The feline enteric coronavirus (FECV) is an ubiquitous enteropathogenic virus, replicating in epithelial cells of both small and large intestine after oral uptake [1-5]. The mild enteritis caused by this replication is usually unapparent or is manifested by a transient diarrhoea in young kittens [3]. Around 13% of all infected cats are not able to clear the virus [6]. In these cats, the virus persists for several months or even years in the epithelium of the large intestine [2-5,7]. Since FECVs are easily transmitted from cat to cat by faecal-oral route, they are enzootic among most cat populations [3,8]. Although FECV-infections manifest subclinically, they may be the start of a lethal outcome. During replication, mutations can occur in the viral genome, providing the virus with tools to productively replicate in monocytes/macrophages [9-12]. This mutational variant, designated feline infectious peritonitis virus (FIPV), causes a chronic and highly fatal systemic disease, FIP, characterized by a diffuse pyogranulomatous (peri)phlebitis and serositis in presence (wet form) or absence (dry form) of fibrinous exudate in the affected body cavities [13-15]. In contrast to FECV which is highly infectious but seldom causes disease, FIPV shows a low infectivity but high mortality (95-100%) [16]. Losses from FIP are typically unpredictable and occur in only a restricted fraction (< 10%) of all seropositive cats [6,16-18]. However, the lack of tools to successfully prevent and control the disease has an enormous financial, emotional and ethical impact, and makes FIP the most feared infectious cause of death in cats [19]. To date, it remains unknown why FECV and FIPV show such a clinically (mild enteritis versus FIP) and epidemiologically (easy versus restricted transmission) different behaviour.

Besides the two pathotypes, FCoVs also occur as two serotypes [20]. Worldwide, the majority of all strains (both FECVs and FIPVs) are serotype I viruses [21-26]. In contrast to the type I viruses that are 100% feline, type II viruses possess spike and adjacent genes of canine origin since they have arisen by double recombination events between type I FCoVs and canine coronavirus (CCV) [27,28]. Despite their lower prevalence, most comparative in vitro studies have been performed with the easily cell culture growing serotype II strains WSU 79–1683 and WSU 79–1146 [9,10,12,29]. FCoV WSU 79–1146 has been shown to be a highly virulent, readily FIP-inducing virus due to its efficient infection of monocytes/macrophages. FCoV WSU 79–1683, on the other hand, is an avirulent virus, inducing at most a mild enteritis in kittens. The poor systemic dissemination of this virus has been attributed to a restricted, inefficient infection of monocytes/macrophages [9,10,12,30]. To date, cell culture propagation of the abundantly present serotype I FECVs has never been achieved and only few serotype I FIPV strains have been adapted to grow in felis catus whole fetus (fcwf) cells. However, most of these strains have lost their pathogenicity through cell culture adaptation [17,31]. Hence, comparative studies between non-culture adapted FECVs and FIPV have only been possible by comparing genomes of both naturally occurring strains [18,32-35]. To date, it remains unclear which genetic determinants make up a certain pathotype.

In the present study, cultures of intestinal epithelial cells from the ileum (ileocytes) and colon (colonocytes) were established by inducing a combined expression of SV40 T-antigen and hTERT in primary ileocytes and colonocytes. The reliability of these cultures for their use in FCoV-research was first investigated by comparing replication capacities of the, at high titre available, avirulent FCoV WSU 79–1683 and the highly virulent FCoV WSU 79–1146 with results obtained for the primary cultures. Since those serotype II strains have been heavily cell culture adapted, the usability of the intestinal epithelial cell cultures in FCoV research was further evaluated by investigating their susceptibility for different field strains, present in faeces and tissues of coronavirus-infected cats.

## Materials and methods

### Cats

Since cats are euthanized every day in practice, tissues of these animals can be used in research in order to reduce the number of laboratory cats. Therefore, the intestines of euthanized conventional cats were used in this study and were a kind contribution to research by the owners. This study was approved by the Local Ethical and Animal Welfare Committee of the Faculty of Veterinary Medicine of Ghent University (EC2012/043) and informed consent was obtained from all owners. Faecal extracts from SPF cats (Harlan laboratories, Indianapolis, IN, USA) experimentally infected with FECV UCD were used as a source of this enteric field strain. These infection experiments were approved by the Local Ethical and Animal Welfare Committee of the Faculty of Veterinary Medicine of Ghent University (EC2012/042).

### Isolation and cultivation of primary ileocytes and colonocytes

Cats were sedated by intramuscular injection of a mixture of Ketamin (0.05 mL/kg; Anesketin®, Eurovet, Heusden-Zolder, Belgium) and Midazolam (0.05 mL/kg; Dormicum**®**, Roche, Brussels, Belgium). Subsequently, the cats were euthanized by intracardial injection of 20% SodiumPentobarbital (1 mL/1.5 kg; Kela Laboratories, Hoogstraten, Belgium). The protocol used for the isolation of primary ileocytes and colonocytes was based on the one described by Rusu et al., with minor adaptations [36]. Directly after euthanasia, the ileum and colon were aseptically removed and transported in ice-cold Dulbecco’s Modified Eagle Medium (DMEM; Gibco BRL, Merelbeke, Belgium) supplemented with 100 U/mL penicillin (Continental Pharma Inc., Puurs, Belgium), 0.1 mg/mL streptomycin (Certa, Braine l’Alleud, Belgium), 0.1 mg/mL gentamycine (Gibco BRL) and 10% foetal bovine serum (FBS; Gibco BRL). Subsequently, the pieces of intestine were inverted, i.e. mucosal side facing outwards, and the intestinal content was removed by three vigorous washings in ice-cold DMEM supplemented with antibiotics. The intestinal mucosa was digested in DMEM containing collagenase I (0.4 mg/mL, Invitrogen, Paisley, UK) and dispase (1.2 mg/mL, Sigma, St. Louis, MO, USA) for 15 min (ileum) or 20 min (colon) at 37 °C. Then, the digestion medium was refreshed and the pieces were incubated for another 45 min (ileum) or 60 min (colon) at 37 °C. Subsequently, the pieces were longitudinally opened and the digested mucosa was scraped with a sterile scalpel blade. The scrapings were incubated in warm DMEM supplemented with antibiotics and dispase (1.2 mg/mL) for 10 min whilst pipetting. After centrifugation (140 × *g*, 3 min) the pellet was resuspended in DMEM containing 2% D-Sorbitol (Sigma) and 10% FBS, and centrifuged (50 × *g*, 3 min) in order to separate as much single cells (most probably contaminating stromal cells) as possible from the epithelial cell clusters. This sorbitol centrifugation was repeated 5 times. The resulting pellet was subsequently resuspended 1:3 (vol:vol) in culture medium consisting of DMEM/F-12 supplemented with 100 U/mL penicillin, 0.1 mg/mL streptomycin and 0.1 mg/mL gentamycin, 10% FBS (Gibco BRL), 10 ng/mL epidermal growth factor (Sigma), 1% insulin-transferrin-selenuim-X (Invitrogen), 100 nM hydrocortisone (Sigma), 1% non-essential amino acids 100× (Gibco BRL), and 1 μg/mL 3,3’ ,5-Triiodo-L-thyronine sodium salt (Sigma). The cells were seeded in 24-well plates or on glass coverslips coated with collagen type I (Roche Diagnostics, Vilvoorde, Belgium). The cells were cultivated in a 37 °C / 5% CO_2_ atmosphere. After 24 h, the culture medium was replaced by medium containing 2% FBS to restrict the outgrowth of non-epithelial cells. Medium was changed every other day. Morphological features of the primary cultures were evaluated every day by light microscopy (Olympus).

### Characterization of the primary cultures

To assess the origin of the primary cells, double-immunostainings were performed against pancytokeratin and vimentin. Therefore, the cells were fixed with 4% paraformaldehyde in PBS for 10 min at room temperature (RT) followed by permeabilization with 0.1% Triton X-100 for 2 min at RT. The cells were incubated with monoclonal anti-cytokeratin antibodies (Dako Denmark A/S) containing 10% normal goat serum for 1 h at 37 °C, followed by goat anti-mouse-Texas Red labelled antibodies for 1 h at 37 °C (Molecular Probes, Eugene, Oregon, USA). Afterwards, the cells were incubated for 45 min at 37 °C with monoclonal anti-vimentin antibodies (Lab Vision Corporation, Fremont, CA, USA) labelled with Zenon® Alexa Fluor 488 (Invitrogen) according to the manufacturer’s protocol. Nuclei were stained with Hoechst 33342 (Molecular Probes) for 10 min at RT. The slides were mounted using glycerine-PBS solution (0.9:0.1, vol:vol) with 2.5% 1,4-diazabicyclo[2.2.2]octane (Janssen Chimica, Beerse, Belgium) and analysed by fluorescence microscopy (DM B fluorescence microscope, Leica Microsystems GmbH, Heidelberg, Germany).

### Immortalization of primary feline ileocytes and colonocytes

At 4 days post isolation, primary cultures of ileocytes and colonocytes from the same cat were transduced with both recombinant lentiviruses expressing either the SV40 large T antigen or the hTERT protein (Applied Biological Materials Inc., Canada) in addition of polybrene (8 μg/mL, Applied Biological Materials Inc.). After 30 min, medium was added and the cells were further incubated with the virus (1:1 vol:vol in medium) overnight. The following day, the viral supernatant was removed and cells were further incubated in medium. After 5 days, the cells were detached by trypsinization with 0.25% trypsin - 0.02% EDTA, subcultured in collagen-coated wells (split ratio 1:2) and evaluated daily for clonal expansion by light microscopy (Olympus). Clusters of cells with epithelial (cobblestone-like) morphology were marked and other cells in the well were removed by scraping. Subsequently, the epithelial clusters were detached by trypsinization and further expanded in collagen-coated flasks to generate a long-term culture of both small and large intestinal epithelial cells.

### Characterization of the ileocyte and colonocyte cell lines

To confirm the epithelial character of both cell lines, double-immunostainings were performed against cytokeratin and vimentin as described above. The success of transduction was assessed by performing immunocytochemical stainings against the SV 40 large T antigen and hTERT. Therefore, cells seeded on collagen-coated glass coverslips were fixed with 4% paraformaldehyde, followed by permeabilization with 0.1% Triton X-100. The cells were incubated with polyclonal rabbit antibodies against hTERT (Applied Biological Materials Inc.) containing 10% normal goat serum for 1 h at 37 °C, followed by goat ant-rabbit-FITC labelled antibodies (Molecular Probes) for 1 h at 37 °C. Subsequently, the cells were incubated with monoclonal antibodies against the SV40 large T antigen (Applied Biological Materials Inc.) containing 10% normal goat serum, followed by goat anti-mouse-AF594 labelled antibodies (Molecular Probes), each for 1 h at 37 °C. Nuclei were stained and slides were mounted as described above. The cells were analysed by fluorescence microscopy (DM B fluorescence microscope, Leica Microsystems GmbH). In addition, immunocytochemical stainings against the intestinal brush border hydrolase aminopeptidase N were performed. Therefore, cells were fixed with 1% paraformaldehyde and incubated with the monoclonal antibody R-G-4 (kindly provided by Dr Hohdatsu, Department of Veterinary Infectious Diseases, Towada, Japan) containing 10% normal goat serum followed by goat anti-mouse-FITC labelled antibodies (Molecular Probes), each for 1 h at 37 °C. Images were obtained using a Leica TCS SPE laser scanning spectral confocal system linked to a DM B fluorescence microscope (Leica Microsystems). Argon and He/Ne lasers were used for exciting FITC and Texas Red fluorochromes, respectively. Leica confocal software was used for image acquisition.

### Expression kinetics of viral antigens in FCoV WSU 79–1683 and FCoV 79–1146 infected cells

A third passage of the FCoV strains 79–1683 and 79–1146 grown in Crandell feline kidney (CrFK) cells were used. FCoV WSU 79–1683 was obtained from the American Type Culture Collection (ATCC) and FCoV WSU 79–1146 was kindly provided by Dr Egberink (Department of Infectious Diseases and Immunology, Utrecht University, the Netherlands). At 4 days post isolation, primary cells of three cats were inoculated at a multiplicity of infection (moi) of 1. After 1 h incubation (37 °C, 5% CO_2_) the cells were washed 3 times with warm DMEM and further incubated in medium. Monolayers of continuous ileocyte and colonocyte cultures were inoculated in the same way. At different time points (0, 3, 6, 9, 12 and 24 h) post inoculation, cells were fixed with 4% paraformaldehyde for 10 min and permeabilized with 0.1% Triton X-100 for 2 min at RT. For the primary cultures, double-immunostainings against both FCoV-antigens and cytokeratin were performed to visualize the infected epithelial cells. For the continuous cultures, only viral antigens were stained. Viral antigens were visualized with polyclonal FITC-labelled anti-FCoV antibodies (VMRD, Pullman, USA). Cytokeratin-positive cells were visualized as described above. Nuclei were stained with Hoechst, the slides were mounted and analysed by fluorescence microscopy (Leica Microsystems GmbH). All experiments were performed 3 times. The area under the curve was determined for each experiment. Triplicate assays were compared using a Mann–Whitney U test. Statistical analysis was performed using GraphPad Prism version 5.0c (GraphPad software, San Diego, CA, USA). *P* values ≤ 0.05 were considered significantly different.

Using primary cells of conventional cats holds the risk that cultured cells are already infected with FCoVs. Therefore, mock-infected cells were accurately screened to exclude the presence of inherent infected cells. All cells were negative for inherent coronavirus.

### One-step real time RT-PCR for the detection of the viral load in field strain suspensions

RNA was extracted from the faecal suspensions using the QIAamp Viral RNA Mini Kit (Qiagen, Benelux BV, Belgium) and from tissue suspensions with the RNeasy Mini Kit (Qiagen). To avoid detection of subgenomic mRNA’s, primers were designed using the Primer 3 plus software within a conserved region of ORF1b based on FCoV sequences available in GenBank. A 20 μL PCR mixture was used per reaction and contained 10 μL Precision OneStep™ qRT-PCR Mastermix with SYBR Green and ROX (PrimerDesign, Southampton, UK), 0.2 μM forward primer ORF1bFW (5’-TGGACCATGAGCAAGTCTGTT-3’), 0.4 μM reverse primer ORF1bRV (5’-CAGATCCATCATTGTGTACTTTGTAAGA-3’) and 3 μL RNA or diluted standard RNA (see below). A reverse transcription step of 10 min at 55 °C and an enzyme activation step at 95 °C for 8 min were followed by 40 cycles, each 10 s at 95 °C and 60 s at 58 °C. A first-derivative melting curve analysis was performed by heating the mixture to 95 °C for 15 s, then cooling to 60 °C for 1 min, and heating back to 95 °C at 0.3 °C increments. Reverse transcription, amplification, monitoring, and melting curve analysis were carried out in a Step One Plus™ real-time PCR system (Applied Biosystems, Life Technologies Corporation, Carlsbad, CA, USA).

### Synthetic RNA standards for absolute quantitation

RNA was extracted from faecal suspensions containing FECV UCD using the QIAamp Viral RNA Mini Kit (Qiagen). The RNA was reverse-transcribed into cDNA using the SuperScript™ III First-Strand Synthesis System for RT-PCR (Invitrogen). Briefly, 250 ng RNA was incubated for 5 min at 65 °C with 2 μM reverse primer ORF1bRV and 10 mM dNTP mix. Afterwards, an equal volume of cDNA synthesis mix, containing 10× RT buffer, 25 mM MgCl_2_, 0.1 M DTT, 40 U/μl RNase OUT and 200 U/μL Superscript III RT was added and incubated for 50 min at 50 °C. The reaction was terminated at 85 °C for 5 min. RNA was removed by incubation with RNase H for 20 min at 37 °C. The 50 μL PCR mixture for the amplification of the cDNA contained 10 μL 5× Herculase II reaction buffer, 0.8 μL dNTP mix, 2 μL DNA template, 0.25 μM forward primer ORF1bFW modified with a T7 promoter sequence at its 5’ end (5’- TAATACGACTCACTATAGGG TGGACCATGAGCAAGTCTGTT-3’), 0.25 μM reverse primer ORF1bRV, and 1 μL Herculase II fusion DNA polymerase (Agilent Technologies Inc., Santa Clara, CA, USA). After a denaturation step for 1 min at 95 °C, 30 cycles of amplification, each 20 s at 95 °C, 20 s at 50 °C, and 60 s at 68 °C, were followed by a terminal elongation of 4 min at 68 °C. Fragment length was controlled by agarose gel electrophoresis and fragments with the correct length were excised and purified from the gel using the Nucleospin® Gel and PCR Clean-up kit (Macherey-Nagel, Düren, Germany). cRNA standards were transcribed by incubation for 1 h at 37 °C with 10× transcription buffer, 500 μM rNTPs and 20 U T7 RNA polymerase-Plus Enzyme Mix (Applied Biosystems). Transcription reactions were DNase I treated and the amount of RNA was determined using the Nanodrop 2000 system. Ten-fold serial dilutions of the RNA were made over a range of 6 log units (10^7^-10^2^) for the generation of the standard curve (Efficiency: 93.96 ± 0.76%; R^2^: 0.999).

### Assessment of the infectious coronavirus titre in faecal and tissue suspensions

Faecal samples were collected from healthy cats housed in 3 different catteries / multi-cat environments that have dealt with FIP in the past. Faecal extracts of experimentally infected cats containing an unknown titre of FECV strain UCD (originally isolated at UC Davis, [3]) were a kind gift of Dr Rottier (Department of Infectious Diseases and Immunology, Utrecht University, the Netherlands). This suspension was clarified by centrifugation at 4000 × *g* for 10 min and SPF cats were infected with the supernatant. Faecal extracts from one cat were used as a source of this enteric field strain. From all faecal samples, 20% suspensions were made in DMEM supplemented with 2% FBS (Gibco, BRL), 100 U/mL penicillin (Continental Pharma Inc.), 0.1 mg/mL streptomycin (Certa), and 0.1 mg/mL gentamycin (Gibco BRL). From 4 cats with FIP (immunohistochemically confirmed), faeces and affected tissues were collected. From tissue homogenates, 20% suspensions were made in DMEM supplemented with 100 U/mL penicillin (Continental Pharma Inc.), 0.1 mg/mL streptomycin (Certa), and 0.1 mg/mL gentamycin (Gibco BRL). Suspensions were centrifuged (1200 × *g*, 4 °C, 20 min), and the supernatant was aliquoted and stored at −70 °C until use. All samples were initially screened by immunofluorescence in both cell lines by inoculating monolayers, seeded on collagen coated coverslips, with 250 μL of the suspensions for 1 h at 37 °C. Thereafter, cells were washed and further incubated in medium for 24 h. After fixation and permeablization, infected cells were visualized as described above. In addition, the amount of infectious virus was quantified in all samples, including the initially negative ones. Therefore, monolayers of colonocytes, seeded in collagen I coated 96-well plates, were inoculated with 50 μL of serially diluted (1/10) faecal or tissue suspensions (ranging from 10^0^ to 10^-7^). After 1 h (37 °C, 5% CO_2_), medium was added and the cells were further incubated for 72 h. To reduce cell loss due to toxicity, undiluted suspensions were removed from the wells 1 h post inoculation (pi), the cells were washed 2 times and further incubated in medium for 72 h. Then, plates were washed with PBS, air-dried (1 h, 37 °C) and frozen (−20 °C). The 50% tissue culture infectious dose (TCID_50_) was determined by means of immunoperoxidase monolayer assay (IPMA). Therefore, cells were fixed and permeabilized by incubation with PF 4% (10 min, RT), followed by incubation with methanol containing 1% H_2_O_2_ (5 min, RT). Then, cells were incubated with PBS containing 10% normal goat serum and 0.1% Tween 80 for 30 min at 37 °C. Subsequently, cells were incubated with monoclonal antibodies against the N-protein (produced and characterized in the laboratory of the authors), followed by incubation with goat anti-mouse HRP-labelled antibodies. Infected cells were visualized by adding sodium-acetate buffer containing amino-ethylcarbazola (AEC) and H_2_O_2_ for 10 min at RT. The fifty percent end-point was calculated according to the method of Reed and Muench [37]. The serotype of all samples was determined by means of RT-PCR described by Addie et al. [22].

### Determination of infectious virus in FIPV-suspensions by inoculation of monocyte-derived macrophages

Feline monocytes were isolated and seeded on glass coverslips as previously described [10]. At 7 days post seeding, cells were inoculated with 250 μL of the suspensions. After 1 h at 37 °C, cells were washed and further incubated in medium for 24 h. After fixation and permeabilization, infected cells were visualized by immunofluorescence staining as described above.

### Propagation and titration of FECV UCD and UG-FH8

Two different faecal strains, UCD and UG-FH8, were passaged 3 times in continuous colonocyte cultures, starting from the faecal suspensions. After 3 passages, the TCID_50_ was determined as described above. In addition, sequencing of ORF3 and ORF7 was performed to check for their integrity. Therefore, primers were designed using published sequences of FCoV ORF3 and ORF7 in GenBank. Viral RNA was extracted from the faecal suspensions with the QIAamp Viral RNA Mini Kit (Qiagen) and cDNA was generated using SuperScript™ III First-Strand Synthesis System for RT-PCR (Invitrogen). Amplification was carried out in a 50 μL reaction using Herculase II fusion DNA polymerase (Agilent Technologies Inc., Santa Clara, CA, USA). The Nucleospin® Gel and PCR Clean-up kit (Macherey-Nagel) was used for purification of the PCR products. Sequencing was performed by the GATC Biotech Company (Konstanz, Germany). Additionally, it was investigated if both third passage strains still showed a specific enterotropism by inoculating other feline cell lines (CrFK and fcwf cells). 24 h pi, infected cells were visualized by immunofluorescence staining as described above.

## Results

### Morphological features and characterization of the primary cultures

**Figure 1:**
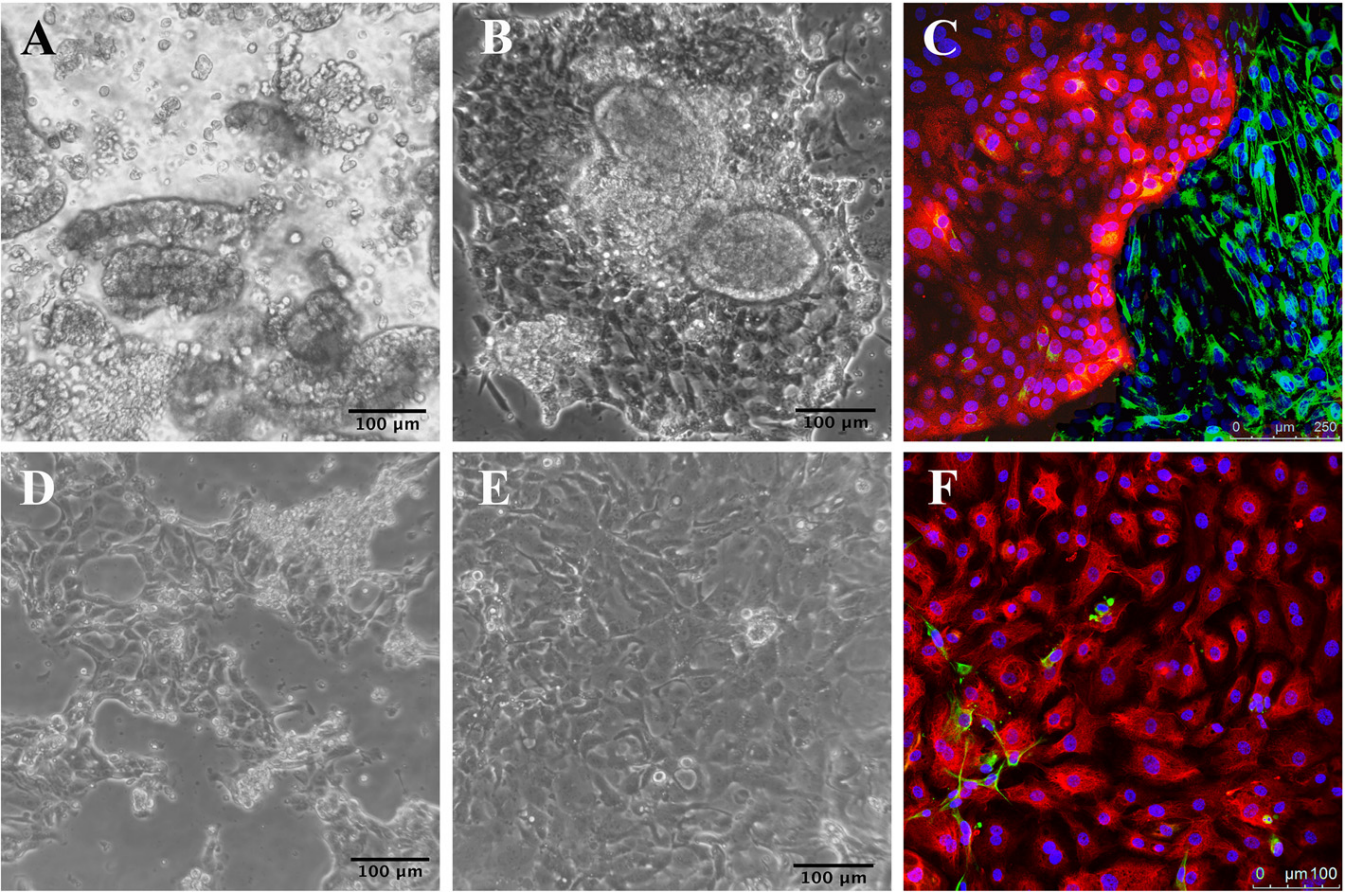
**Morphological features and immunocytochemical characterization of the primary ileum (A-C) and colon (D-F) cultures. (A)** Epithelial cells were isolated in cell clusters. **(B, D)** Polygonal cells started to spread from these clusters giving rise to several foci of cells. **(E)** (Sub) confluent layers were reached 3–4 days after seeding due to a restricted proliferation of the cells. **(C, F)** Double-immunostainings against cytokeratin (red) and vimentin (green) filaments 4 days after isolation, confirming the epithelial nature of the polygonal cells.

By using a combination of dispase and collagenase, epithelial cells were isolated from the underlying basement membrane in clusters (Figure 1A). Four hours post seeding, the majority of the cells had attached and foci of polygonal cells became visible within 24 h post seeding (Figure 1B, 1D). Primary ileum cultures were always “contaminated” with a lot of elongated or stellate-like cells, present in between the epithelial foci, while the colon cultures were more pure. For the ileum, the epithelial cells did not further grow beyond 24 h post seeding, whereas mesenchymal cells started to expand in between the epithelial cell clusters. In the colonic cultures, the epithelial cells showed a confined proliferation within 3–4 days post isolation, resulting in the formation of (sub)confluent cobblestone-like layers (Figure 1E). Then, these cells had reached a state of replicative senescence, which became typically characterized by morphological changes such as increase in cell size and development of multiple nuclei at 6–7 days post isolation. The growth arrest seemed not to be the result of the confluent state since, despite many attempts, it was not possible to subculture the cells. A part of the cells started to degenerate from 7 days post seeding. However, most of the cells could be kept for another week. To prevent cell loss due to inherent degeneration and to prevent overgrowth by mesenchymal cells, both ileum and colon cultures were always infected at 4 days post isolation for studying the viral replication.

Immunofluorescence stainings against cytokeratin (intermediate filaments typically found in the cytoskeleton of epithelial cells) and vimentin (intermediate filaments expressed by mesenchymal cells) confirmed the epithelial nature of the polygonal, cobblestone-like cells (Figure 1C, 1F). At 4 days post isolation, the majority of the cells (> 90%) in the colon cultures was still of epithelial origin. For the ileum cultures, the vimentin positive mesenchymal cells had expanded in between the epithelial clusters, occupying around 50% of the wells. Remarkably, some of the ileum epithelial cells did also express vimentin, resembling dedifferentiated epithelial cells typically found after injury or in tumours (Figure 1C).

### Expression kinetics of viral antigens in FCoV WSU 79–1683 and WSU 79-1146-infected primary ileocytes and colonocytes

**Figure 2:**
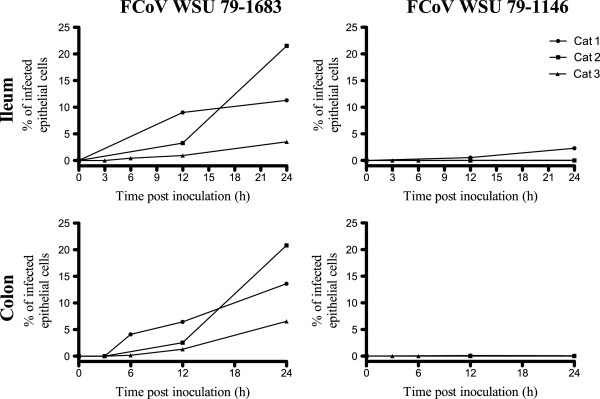
**Kinetics of FCoV replication in primary ileum and colon cultures from 3 conventional cats.** Cells were inoculated with FCoV WSU 79–1683 or FCoV WSU 79–1146 at a moi = 1. At different time points post inoculation, cytoplasmically expressed viral proteins were visualized and the percentage of infected epithelial cells was determined.

Primary ileocytes and colonocytes were susceptible to infection with both serotype II FCoV strains. However, the antigen expression kinetics differed greatly between the avirulent FCoV WSU 79–1683 and the virulent FCoV WSU 79–1146 (Figure 2). For both strains, the first antigen-positive cells appeared at 6 h pi and increased further over time. However, the avirulent enterotropic WSU 79–1683 strain infected the cells significantly more efficient (*P* = 0.05 for both ileum and colon) compared to WSU 79–1146.

### Morphological features and characterization of the established continuous ileocyte and colonocyte cultures

**Figure 3:**
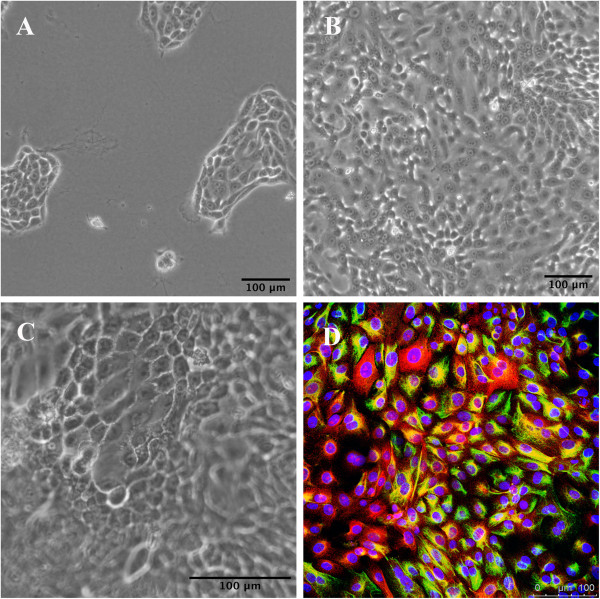
**Morphological and immunocytochemical characterization of the continuous ileocyte cultures. (A)** Proliferating isles. **(B)** Cobblestone morphology of the monolayer. **(C)** Dome formation. **(D)** Double-immunostaining against cytokeratin (red) and vimentin (green) filaments.

**Figure 4:**
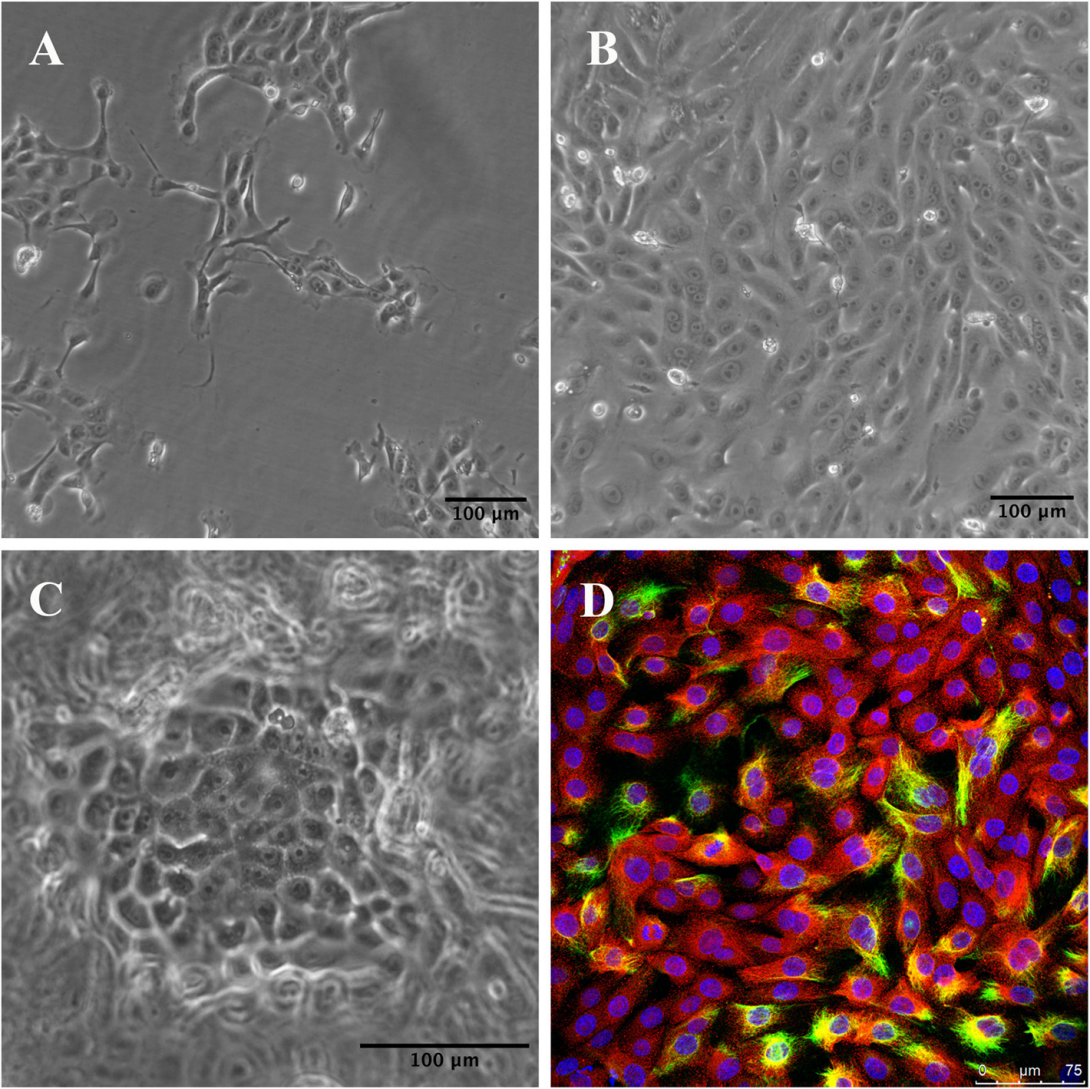
**Morphological and immunocytochemical characterization of the continuous colonocyte cultures. (A)** Proliferating isles. **(B)** Cobblestone morphology of the monolayer. **(C)** Dome formation. **(D)** Double-immunostaining against cytokeratin (red) and vimentin (green) filaments.

By introducing a combinational expression of SV40 large T-antigen and hTERT, a successfully transformed cell line was generated for both ileocytes and colonocytes (Figures 3 and 4). Indeed, a various number of the transduced cells started to proliferate from 1 week after transduction onwards, forming layers of cobblestone-like cells with a cell diameter of 20–25 μm and 30–35 μm for ileocytes and colonocytes, respectively. Both SV40 large T-antigen as hTERT expression was detected in these cultures, confirming the success of transduction. These cell lines could be further expanded and passaged for over 30 passages now, which is in sharp contrast to the primary cultures. Besides its typical cobblestone-like appearance, the epithelial character was confirmed by the expression of cytokeratin and dome formation in the cultures. The latter is indicative for the polarization of cells in monolayers. Remarkably, most of the cells in both cultures co-expressed both cytokeratin and vimentin in the freshly formed monolayers, suggesting a more dedifferentiated state of the cells. For further characterization, APN expression in the cultures was investigated, since APN is an intestinal brush border associated hydrolase, and moreover an important receptor for serotype II FCoVs. All cells expressed APN at their surface. However, the expression levels varied great from cell to cell in both cultures, most probably due to different differentiation levels of the cells in culture.

### Antigen expression kinetics of FCoV WSU 79–1683 and WSU 79–1146 in continuous ileocyte and colonocyte cultures

**Figure 5:**
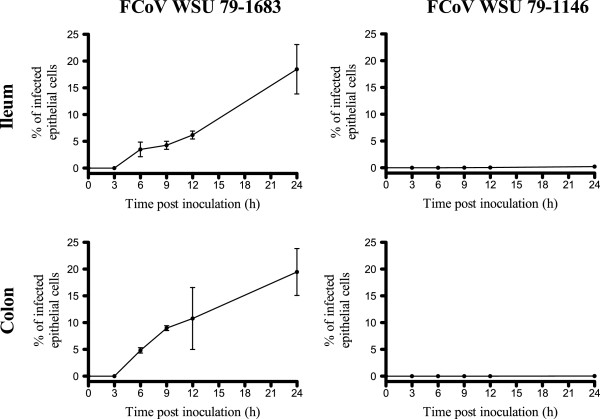
**Kinetics of FCoV replication in continuous ileocyte and colonocyte cultures.** Cells were inoculated with FCoV WSU 79–1683 or FCoV WSU 79–1146 at a moi = 1. At different time points post inoculation, the percentage of infected cells was determined. Data are expressed as the means ± standard deviation of the results of 3 separate experiments.

Since the continuous cultures seemed to be less differentiated compared to the primary cultures, the reliability of the established cell lines as model for intestinal epithelial cells was further investigated. Therefore, antigen expression kinetics were assessed in both continuous ileocyte and colonocyte cultures as was done for the primary cells (Figure 5). In accordance with the results obtained for the primary cultures, FCoV WSU 79–1683 significantly infected both ileocytes as colonocytes more efficiently than WSU 79–1146. At 24 h pi, FCoV WSU 79–1683 had infected 19.46 ± 4.37% and 18.47 ± 4.61% of the colonocytes and ileocytes, respectively, whereas only 0.03 ± 0.02% of the colonocytes and 0.22 ± 0.18% of the ileocytes were infected by FCoV WSU 79–1146 at that time point.

### Titration of field strains in faecal and tissue suspensions

A major restriction in FCoV research is the lack of cell lines supporting the growth of serotype I enteric strains. Therefore, the newly established cell lines were further validated by investigating their susceptibility for different field strains. All those strains were serotype I viruses as confirmed by PCR. Table 1 gives the results obtained by titration of different faecal and tissue suspensions on colonocyte cultures. Comparable results were obtained by titration on ileocyte cultures with FECV UCD. Hence, titration of other field strains was not repeated on this cell line. All but two of the samples collected from healthy cats were positive for coronavirus, with qPCR titres ranging from 10^4.18^ to 10^9.06^ viral copies / g faeces. Infectious virus was detected by IPMA in 50% of all positive samples (8/16), with 57% of positivity in samples with qPCR titres above 10^5^. This number increased to 64% (7/11) and 80% (4/5) when the cutoff was made at qPCR titres above 10^6^ and 10^7^ viral copies / g faeces, respectively. In the one sample (UG-FH9) with a qPCR above 10^7^ that was negative on IPMA, enterotropic virus was detected by immunofluorescence staining. All but one of the samples collected from FIP cats were positive for coronavirus on qPCR, with the number of viral copies / g ranging from 10^3.98^ to 10^9.16^. As determined by both IPMA and immunofluorescence staining, none of those samples, except for one, contained enterotropic virus. However, 3 tissue samples (UG-TF5, UG-TF9 and UG-TF17) did contain infectious virus as determined on monocyte-derived macrophages. Despite its high viral load, no infectious virus (neither on enterocytes nor on monocytes/macrophages) was found in faecal suspensions of FIP cat 1 (UG-FF1). Faeces of FIP cat 2 (UG-FF2) did contain enterotropic virus that was not infectious for macrophages.

**Table 1:** QPCR- and infectious titre of different faecal and tissue suspensions from healthy and FIP cats

**Sample**	**Source**	**QPCR titre (Log**_**10 **_**copies / g)**	**Infectious titre (Log**_**10 **_**TCID**_**50 **_**/ g)**
UG-FH1	Faeces healthy cats	6.03	-
UG-FH2	Faeces healthy cats	6.64	2.67
UG-FH3	Faeces healthy cats	5.51	-
UG-FH4	Faeces healthy cats	5.41	2.36
UG-FH5	Faeces healthy cats	7.22	2.50
UG-FH6	Faeces healthy cat	6.88	-
UG-FH7	Faeces healthy cat	-	-
UG-FH8	Faeces healthy cat	6.30	3.33
UG-FH9	Faeces healthy cats	7.69	-
UG-FH10	Faeces healthy cat	7.89	2.50
UG-FH11	Faeces healthy cats	8.44	2.67
UG-FH12	Faeces healthy cats	4.66	-
UG-FH13	Faeces healthy cats	-	-
UG-FH14	Faeces healthy cats	6.27	-
UG-FH15	Faeces healthy cat	6.62	2.50
UG-FH16	Faeces healthy cats	4.18	-
FECV UCD	Faeces healthy cat 6d pi	9.06	5.00
UG-FF1	Faeces FIP cat 1	7.57	-
UG-FF2	Faeces FIP cat 2	9.16	3.50
UG-FF3	Faeces FIP cat 3	-	-
UG-FF4	Faeces FIP cat 4	3.98	-
UG-TF2	Kidney FIP cat 1	6.79	-
UG-TF5	Omentum FIP cat 2	6.87	-
UG-TF9	Spleen FIP cat 3	5.83	-
UG-TF17	Omentum FIP cat 4	8.00	-

### Propagation and titration of FECV UCD and UG-FH8

**Figure 6:**
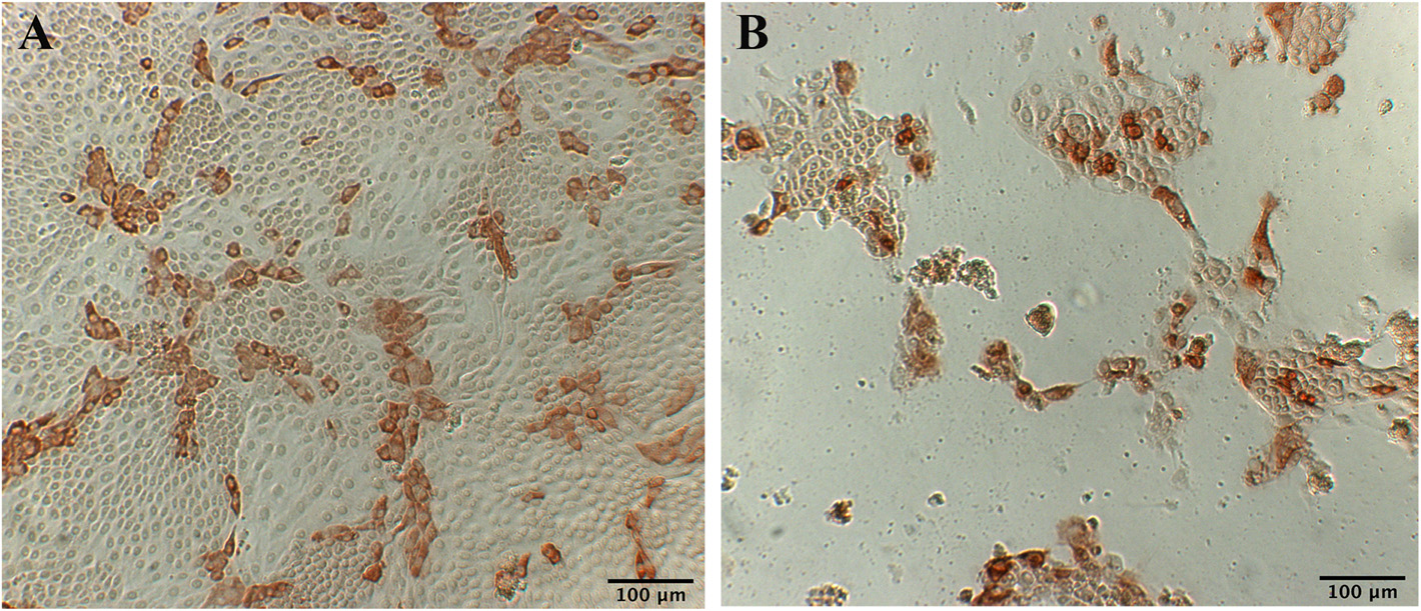
**Immunoperoxidase staining of infected colonocytes.** Infected colonocytes 3 days pi with **(A)** 10^2.99^ TCID_50_ FECV UCD and **(B)** 10^2.67^ TCID_50_ UG-FH8.

To date, no serotype I enteric field strains have been propagated in vitro and availability of such FECV strains would be valuable in feline coronavirus research. Therefore, two faecal strains, FECV UCD and UG-FH8, were further propagated in colonocyte cultures (Table 2). After 3 passages, both strains were raised in titre with around 3 log_10_ TCID_50_ / mL. In addition, ORF3 and ORF7 from each of the third passage strains were sequenced to check for signs of cell culture adaption. Both strains still carried intact accessory genes that were 100% identical to the original strain. Typically, a lot more CPE was noticed in UG-FH8 infected wells compared to FECV UCD (Figure 6). After 3 passages, both strains still showed a specific enterotropism, since no infection was seen after inoculation of other feline cell lines (fcwf and CrFK cells).

**Table 2:** Infectious titre and status of ORF3 and ORF7 in cell culture propagated viruses

**Strain**	**Infectious titre (Log**_**10 **_**TCID**_**50 **_**/ mL)**		**Status ORF3 at P**_**3**_	**Status ORF7 at P**_**3**_
	**P**_**0**_	**P**_**3**_		
FECV UCD	3.97	6.30	Intact	Intact
UG-FH8	2.63	5.97	Intact	Intact

## Discussion

In this study, immortalized cultures of both small (ileum) and large (colon) intestinal epithelial cells were established and validated for their use in feline coronavirus research. Intestinal epithelial cells are important target cells in FCoV pathogenesis but to date such cell lines are not available. The establishment of primary intestinal epithelial cell cultures has been proven to be difficult because of the induction of programmed cell death after disruption from the extracellular matrix, the uncontrolled contamination with stromal cells and the still unknown homeostatic components needed for the maintenance of these cultures [38]. To avoid induction of apoptotic signals by disrupting cell-matrix adhesions, a combination of collagenase and dispase was used in this study to digest the mucosa, allowing the isolation of epithelial cell clusters. These were subsequently separated as much as possible from the contaminating single stromal cells by D-sorbitol density centrifugation. The primary colon cultures showed a relative high purity of epithelial cells, whereas primary ileum cultures were much more contaminated with stromal cells. The contamination with mesenchymal cells is intrinsic to the isolation method used and therefore inevitable. Yet, the epithelial cells could be cultured for a week without overgrowth by these cells, making both primary cultures ideal models for studying interactions with enterotropic infectious agents. Remarkably, some primary cells co-expressed cytokeratin and vimentin filaments, which is often found in injured epithelial cells, tumours and in primary cultures due to the detachment of the cells from their natural environment during isolation. In these cells, the epithelial differentiation is turned back to a more embryonic state, amongst others characterized by de novo expression of vimentin filaments [39]. Only a minority of the cells did express vimentin, suggesting that most cells were able to restore their differentiation with the used culture conditions.

Although the doubtful origin and clear signs of cell culture adaptation [17,40], FCoV WSU 79–1683 and FCoV WSU 79–1146 were the only available strains representing an avirulent and related virulent strain at that time of the study. Hence, those strains were initially used for investigating the susceptibility of primary enterocytes to both virulent and avirulent FCoVs. Replication of both strains have been studied in CrFK cells, fcwf cells, peritoneal macrophages, bone marrow-derived macrophages and peripheral blood monocytes [9,10,12,29]. In contrast to the available continuous cultures (CrFK and fcwf cells), the difference in virulence between both strains was reflected in vitro when using primary FIPV target cells (monocytes/macrophages). The highly efficient and mostly sustained infection of FIPV in macrophages and monocytes from susceptible cats, in contrast to an inefficient and not sustained infection of the avirulent WSU 79–1683 in those cells, may explain why FIPV behaves as a harmful invasive virus causing this progressive systemic disease [9,10,12]. As was previously shown for monocytes/macrophages, the present study confirms that both strains exhibit clear differences in cell tropism. In contrast to FCoV WSU 79–1146, the avirulent WSU 79–1683 efficiently infected and replicated in intestinal epithelial cells, resulting in exactly opposing kinetics as were found for macrophages [9].

Primary cultures are ideal tools to reliably investigate virus-host interactions. Nevertheless, isolation of primary epithelial cells is labour-intensive, the cultures are often contaminated with a various amount of mesenchymal cells and the yield is variable and rather low. To activate research with those cells, long-term cultures were derived from both primary ileocytes and colonocytes by SV40 T-antigen- and hTERT-induced immortalization, resulting in the generation of two feline intestinal epithelial cell cultures. The epithelial nature of both cell lines was confirmed by their cobblestone morphology, dome formation and cytokeratin expression. These newly established cell lines could be valuable tools for virus research. However, immortalized cell lines are often phenotypically transformed, making reliable research with these cells questionable. In the present study it was shown that, in contrast to the primary cultures, the majority of the cells co-expressed cytokeratin and vimentin filaments, suggesting that the cultures were less differentiated compared to their primary counterparts. Therefore, the reliability of the established cell lines for their use in feline coronavirus research was further investigated and confirmed. Antigen expression kinetics of FCoV WSU 79–1683 and FCoV WSU 79–1146 were comparable with the results obtained with the primary cultures, showing a significant difference in cell tropism between both strains. As mentioned before, comparative studies in the available continuous feline cell lines (CrFK and Fcwf cells) showed no replicative differences between both serotype II strains [9,10,29]. However, both cultures are hardly sensitive to serotype I FCoVs. To date, cultivation of serotype I FECVs has never been achieved and only few serotype I FIPV strains could be adapted to grow in continuous cell cultures. In addition, most of these strains seem to have lost their pathogenicity through cell culture adaptation [17,31]. In the present study, the newly established intestinal epithelial cell cultures were further evaluated for their susceptibility to serotype I field strains. Infectious, enterotropic virus was found in 57% (8/14) of all FCoV-positive faecal samples originating from healthy cats in 3 geographically distinct multi-cat environments. One of those samples was detected only by immunofluorescence staining. This higher sensitivity can be explained by the use of more inoculum in that test. In the majority of the positive samples, infectious titres were always between 10^3.05^ to 10^5.77^ times lower compared to the total virus titre. This difference can be attributed to the presence of defective particles, but infectious titres in such faecal samples can possibly be underestimated due to faecal toxicity to the cells and the presence of neutralizing IgA antibodies as well. In infection experiments with FECV UCD, the amount of infectious particles was typically 3–4 log_10_ times lower compared to the total amount of particles in the first week pi, but this further increased thereafter most probably due to the generation of neutralizing antibodies (data not shown). It is impossible to estimate when cats in multi-cat environments became infected and the presence of neutralizing antibodies can explain why infectious virus in some of the faecal samples with a quite high viral load was not detectable. Coronavirus was detected in 3/4 of the tested faecal samples from FIP cats. Previously, it has been shown that faecal viruses from FIP cats did not cause enteric infections or FIP upon inoculation of laboratory cats [18]. This can explain why, despite its high viral load, no infectious virus (neither in enterocytes nor in monocytes/macrophages) was found in the faeces of FIP cat 1 (UG-FF1). However, enterotropic virus was found in the faeces from another FIP cat (UG-FF2) that was housed in a Belgian shelter. To search for explanations for this discrepancy, accessory proteins of the virus in faecal and tissue suspensions of that cat were sequenced (data not shown). As in all faecally shed FCoVs sequenced so far [18,32,35], the faecal strain carried an intact 3c gene. In addition, this strain showed only 96% and 89% homology with the tissue strain based on 7a and 7b protein respectively. So it seems that this cat was co-infected with another, most probably enteric strain circulating in that shelter, explaining the shedding of enterotropic infectious virus in that cat. In 3/4 of the tissue samples from FIP cats (UG-TF5, UG-TF9 and UG-TF17), infectious virus was found by inoculation of monocyte-derived macrophages. However, these viruses seemed to have lost their tropism for intestinal epithelial cells since no infection was detected after inoculation of the intestinal epithelial cell cultures. The fact that FECV is the only pathotype that is well adapted for growth in intestinal epithelial cells shows that FECVs have the advantage over FIPVs to spread amongst cats. These findings are in agreement with previous observations on FCoV epidemiology, explaining the restricted transmission of FIPVs and hence low incidence of cats with FIP [6,16-18].

Since no cell culture-propagated serotype I enteric strains are available, two of those strains, FECV UCD and UG-FH8, were further propagated in the established cultures. After 3 passages, both virus strains were raised in titre with 3 log_10_ TCID_50_ / mL, making them usable for further in vitro experiments. It has been described that the 7b glycoprotein is not necessary for replication in cell cultures and hence this gene is readily lost by in vitro propagation. Therefore, alterations in the 7b protein can be a sign for cell culture adaptation as seen in many of the cell culture propagated serotype I FIPVs [41]. In present study, no such signs of cell culture adaptation were detected for both 3^th^ passage strains, which still carried intact ORF7 genes identical to the original faecal strains. All field enteric strains sequenced so far carried intact 3c genes [18,32,35]. To date, the only available avirulent, enteritis-inducing strain, WSU 79–1683, has a mutated 3c gene and for that reason doubt has been cast on the use of this strain as a typical enteric pathotype [17,40]. Both FECV UCD and UG-FH8 propagated in this study still carried an intact (and identical to the original strain) ORF3. In addition, the cell culture propagation of both strains did not extend their tropism to other non-enterocytic feline cells, making them useful as representatives of the enteric pathotype.

In conclusion, we established cultures of both feline small and large intestinal epithelial cells, providing new and reliable in vitro models for studying enteric pathogenesis processes of FCoVs. These enterocyte cultures were susceptible to different enteric serotype I field strains, while FIPVs were clearly restricted in their replication in intestinal epithelial cells. Two of the enteric strains were further propagated, providing relevant enteric strains for future FCoV research.

## Competing interests

The authors declare that they have no competing interests.

## Authors’ contributions

LMBD participated in the design of the study, carried out cell isolation, characterization and immortalization processes, performed and analysed infection studies and drafted the manuscript. ST participated in the design and performance of cell isolation, characterization and real time RT-PCR procedures. DAJO participated in cell characterization and immortalization. AD helped in the design of real time RT-PCR procedures. BLV participated in statistical analysis and infection experiments. IDMR contributed in sequencing studies. HJN designed and coordinated the study, and contributed in the interpretation of data and the final version of the manuscript. All authors read and approved the final manuscript.
